# Supplementary material for: Analysis of Conservation Priorities and Runs of Homozygosity Patterns for Chinese Indigenous Chicken Breeds
Source: Animals (Basel). 2023 Feb 8;13(4):599. doi: 10.3390/ani13040599 (PMC9951684; doi:10.3390/ani13040599)
Supplement: Supplementary file 1 [file animals-13-00599-s001.zip › Table S1. Breeds information.pdf]

Table S1. Breeds information.

Breeds	Label	Number	local	Specific features	Origin of sample
Baier Chicken	Baier	18	Shangrao city, Jiangxi	Light-sized, three yellow, layer	[1]
Chahua Chicken	Chahua	19	Xishuangbanna, Yunnan	Light-sized, meat and egg dual-purpose breed	[1]
Figthing Chicken (Henan game)	Figthing	20	Zhengzhou city, Henan	Heavy-sized of purpose breed, fancy breed	[1]
Gushi Chicken	Gushi	20	Gushi county,Henan	Medium-sized, Three yellow, meat and egg dual-purpose breed	[1]
Langshan Chicken	Langshan	20	Zhengzhou city, Henan	Heavy-sized, meat and egg dual-purpose breed	[1]
Wannan three-yellow chicken	WanTy	20	Qinyan county, Anhui	Medium-sized, three yellow,, egg purpose breed	[1]
Wugu Chicken	Wugu	20	Taihe county,Jiangxi	Light-sized, White feather, black skin, black bone	[1]
Xiaoshan Chicken	Xiaoshan	20	Taihe county,Jiangxi	Heavy-sized, meat and egg dual-purpose breed	[1]

*three yellow features (plumage yellow, beak yellow and shank yellow)

The image of 8 Chinese indigenous chicken breeds:

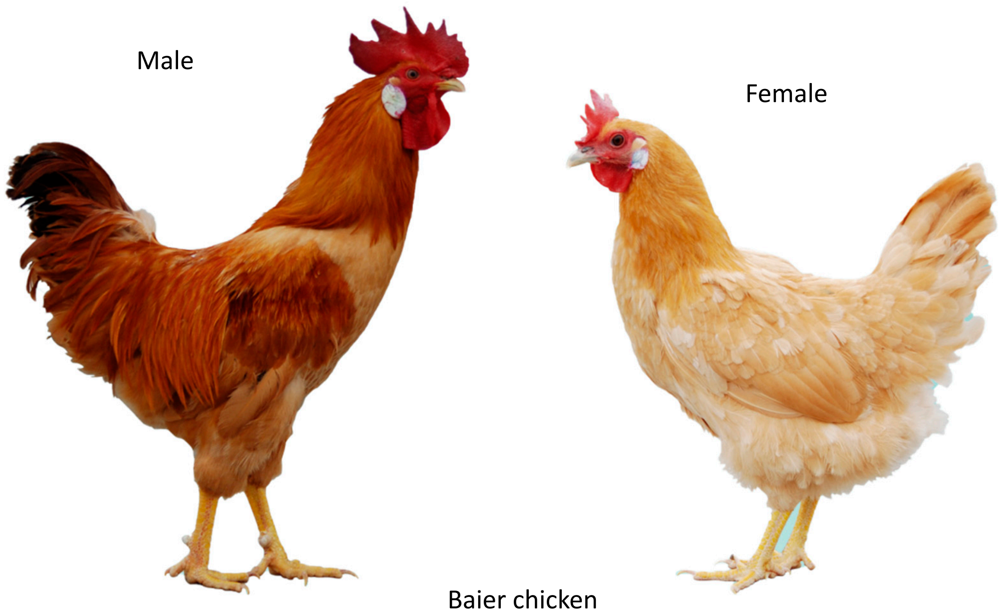

Baier chicken

Male

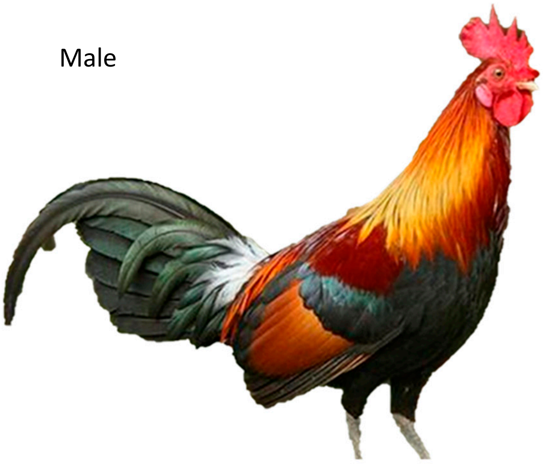

Female

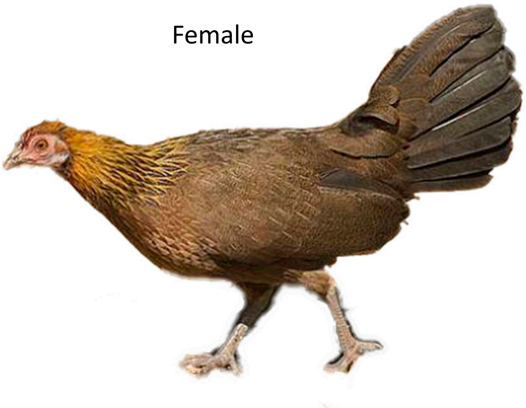

Chahua chicken

Male

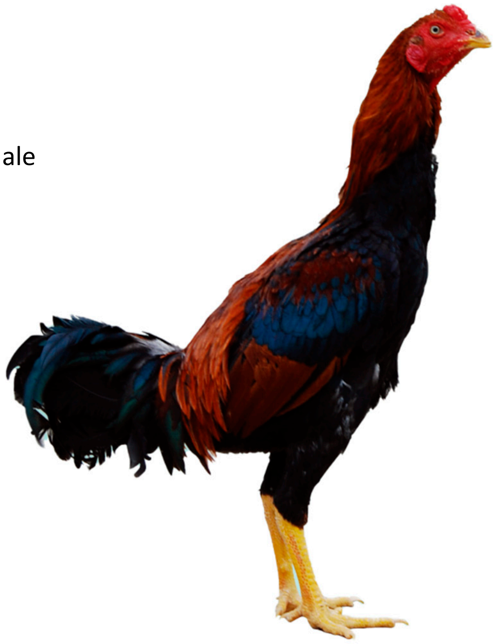

Female

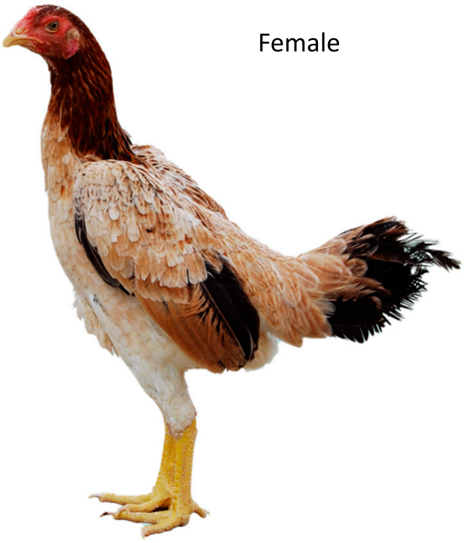

Fighting chicken

Male

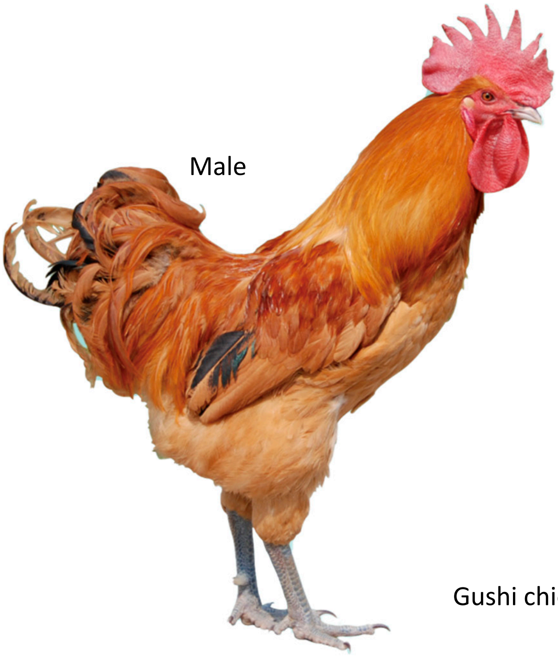

Famale

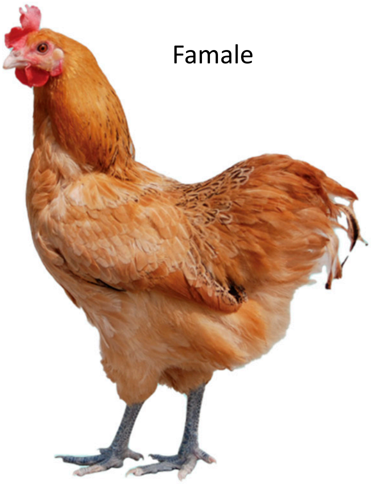

Gushi chicken

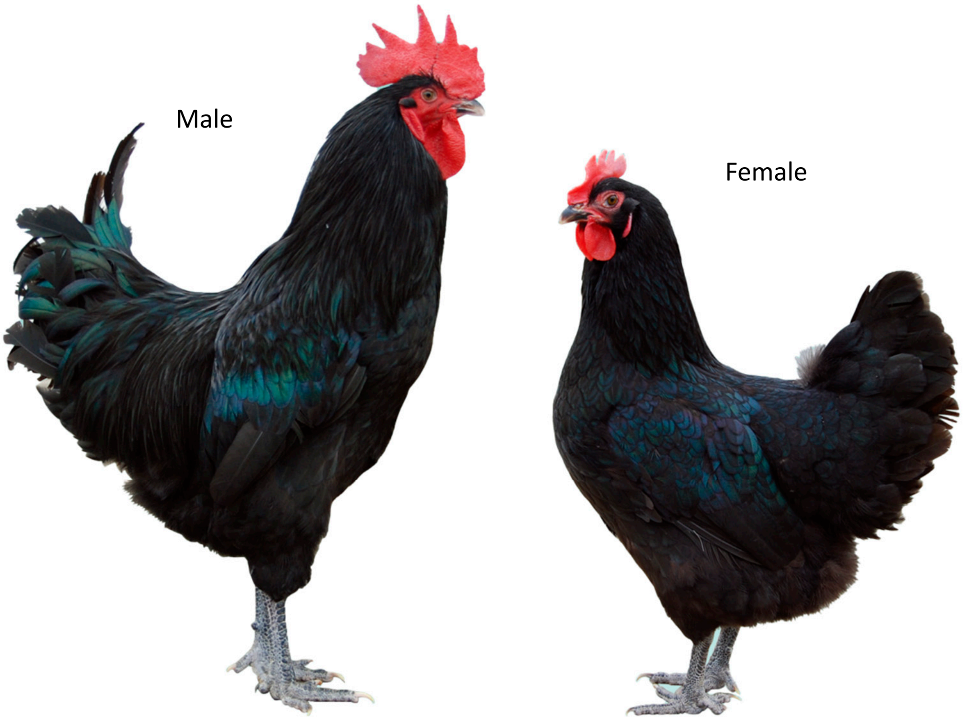

Langshan chicken

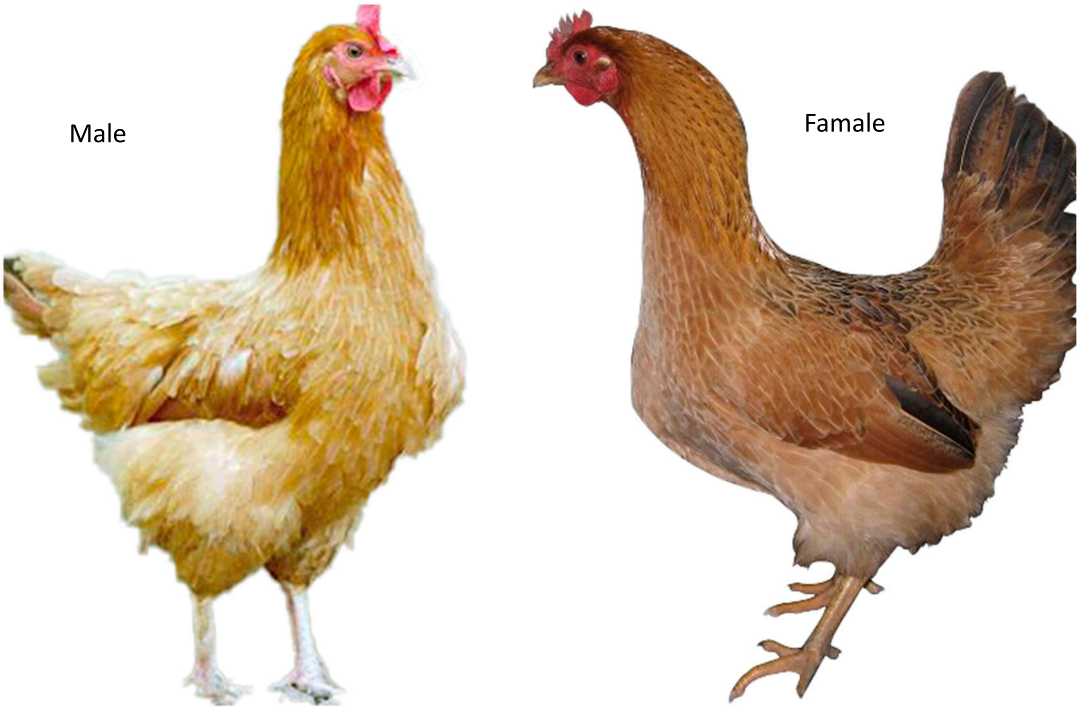

Wannan three-yellow chicken

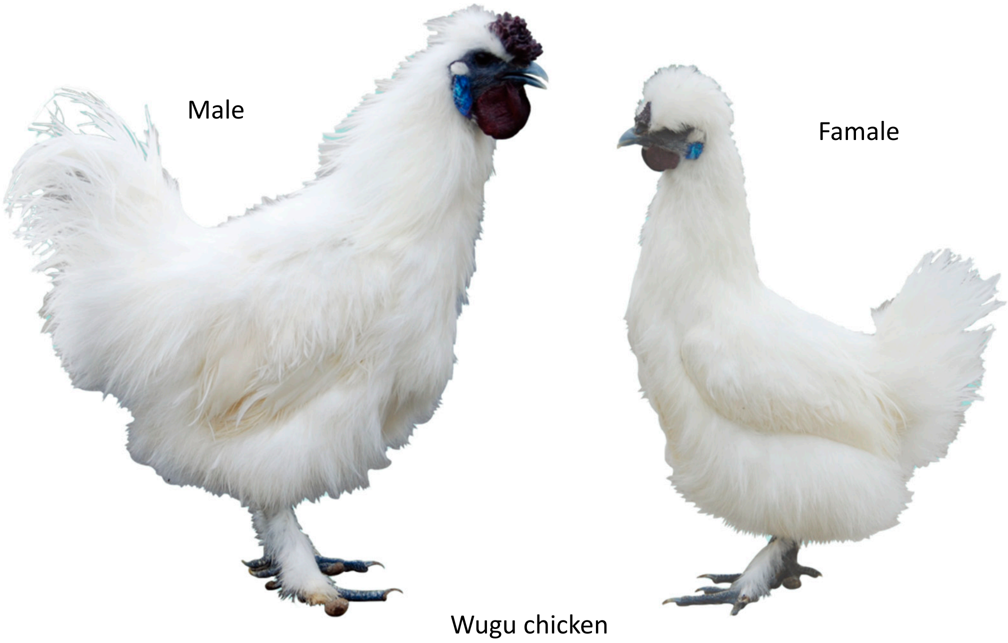

Wugu chicken

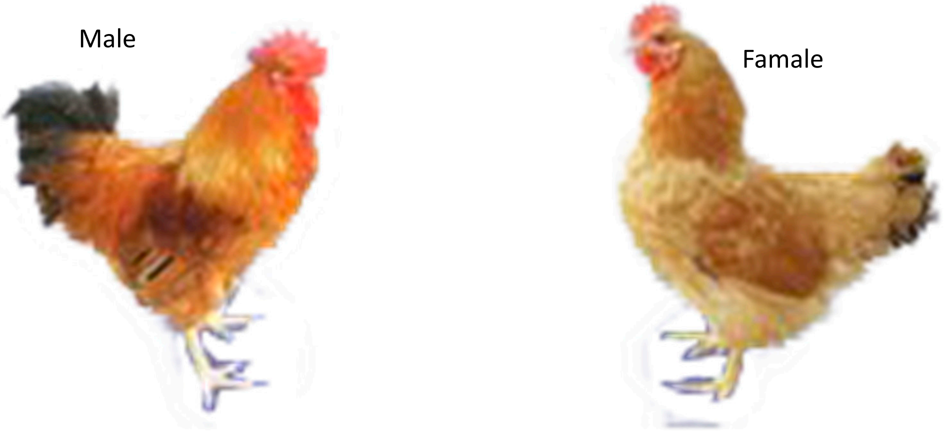

Xiaoshan chicken

Refence

1. Malomane, D. K.; Simianer, H.; Weigend, A.; Reimer, C.; Schmitt, A. O.; Weigend, S., The SYNBREED chicken diversity panel: a global resource to assess chicken diversity at high genomic resolution. *BMC genomics* **2019,** *20*, 1-15.
